# Small-molecule induction of phospho-eIF4E sumoylation and degradation via targeting its phosphorylated serine 209 residue

**DOI:** 10.18632/oncotarget.3615

**Published:** 2015-04-10

**Authors:** Ying Gu, Hong Zhou, Yichao Gan, Jiawei Zhang, Jianghua Chen, Xiaoxian Gan, Hongzhi Li, Weiwei Zheng, Zhipeng Meng, Xiaoxiao Ma, Xichun Wang, Xiaohua Xu, Ganyu Xu, Xiaoya Lu, Yun Liang, Xuzhao Zhang, Xinliang Lu, Wendong Huang, Rongzhen Xu

**Affiliations:** ^1^ Department of Hematology, Key Laboratory of Cancer Prevention and Intervention, China National Ministry of Education, Second Affiliated Hospital, School of Medicine, Zhejiang University, Hangzhou 310009, China; ^2^ Cancer Institute, Second Affiliated Hospital, School of Medicine, Zhejiang University, Hangzhou 310009, China; ^3^ Division of Molecular Diabetes Research, Department of Diabetes and Metabolic Diseases Research, Beckman Research Institute, City of Hope National Medical Center, Duarte, CA 91010, USA; ^4^ Zhejiang Academy of Medical Sciences, Hangzhou 310012, China; ^5^ Department of Molecular Medicine, Beckman Research Institute, City of Hope National Medical Center, Duarte, CA 91010, USA

**Keywords:** phospho-eIF4E, homoharritonine, small molecular inhibitor, proteasome-dependent degradation, acute myeloid leukemia

## Abstract

As phospho-eIF4E (p-eIF4E), unlike total eIF4E (t-eIF4E) essential for normal cells, is specifically required by cancer cells, it is an attractive, yet unrealized, target for anti-tumor intervention. Here we identify a small molecule, homoharringtonine (HHT), that antagonizes p-eIF4E function and eradicates acute myeloid leukemia (AML) expressing high level of p-eIF4E *in vitro* and *in vivo*. HHT selectively reduces p-eIF4E levels of leukemia cells without affecting t-eIF4E. HHT targets the phosphorylated serine 209 residue of p-eIF4E and induces p-eIF4E oligomerization, which enhances its interaction with the small ubiquitin-like protein modifier (SUMO)-conjugating enzyme UBC9, resulting in proteasome-dependent degradation of p-eIF4E via SUMO2/3-mediated SUMOylation. These results suggest that the phosphorylated serine 209 residue of p-eIF4E might be a potential target for developing small molecule-based new therapies for leukemia.

## INTRODUCTION

Acute myeloid leukemia (AML), the most common acute leukemia in adults, is a heterogeneous group of clonal disorders characterized by an abnormal proliferation of myeloid stem/progenitor cells and subsequent bone marrow (BM) failure (1–4). Although a high proportion of AML patients can achieve complete remission by current chemotherapies, most of these patients ultimately relapse (5, 6). Hence, there is an urgent need for more potent and safer therapies against leukemia-associated targets for AML.

Phospho-eIF4E (p-eIF4E), unlike eIF4E that is essential for survival and growth of normal cells (7–9), is specifically required by cancer cells including AML cells (10–16). Consistent with this, studies have shown a positive correlation between increased eIF4E phosphorylation and cell proliferation (17, 18), and highly phosphorylated eIF4E was observed in a variety of cancers (10, 19). In particular, recent studies have shown that inhibiting p-eIF4E with small molecule inhibitors of MNK that phosphorylates eIF4E, exhibited a potent inhibition for leukemia (19, 20). By contrast, the nonphosphorylatable form of eIF4E has been shown to be not sufficient for transformation and even resistant to tumorigenesis in a prostate cancer model (10). Thus, we reasoned that small molecules that directly target the p-eIF4E but not eIF4E would exhibit more potent and safer therapeutic effects for cancer.

eIF4E plays a critical role in exporting of mRNAs of pro-oncogenic or pro-survival proteins having short half-lives, such as Mcl-1, cyclin-D1 or c-Myc (7). Coincidentally, studies have shown that homoharringtonine (HHT), a natural alkaloid that exhibit potent effective for patients with AML-M5 (21), selectively inhibited the synthesis of these short half-live oncoproteins (22–24). These data, together with our recent findings that p-eIF4E is highly upregulated in AML-M5 patients (Supplementary Fig. S1A–B), attempted us to reason that HHT may inhibit growth of AML cells via directly targeting p-eIF4E.

## RESULTS

### HHT specifically reduces p-eIF4E and p-eIF4E is a critical target for its anti-leukemia activity

**Figure 1:**
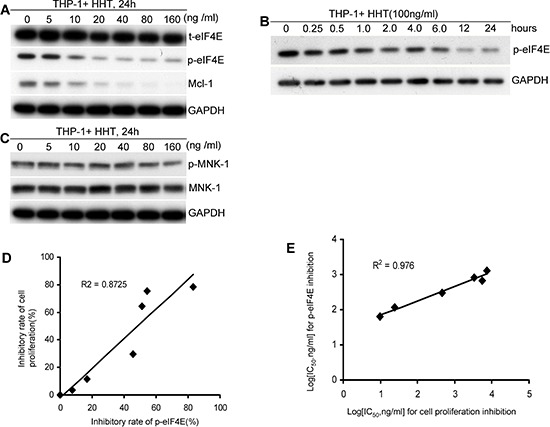
HHT specifically reduces p-eIF4E and p-eIF4E is a critical target for its anti-leukemia activity **A.** HHT specifically reduced p-eIF4E and its downstream molecule levels of leukemia cells in a dose-dependent manner but did not alter t-eIF4E. **B.** HHT reduced p-eIF4E level of leukemia cells in a time-dependent manner. **C.** HHT did not alter phospho- and total-Mnk1 levels of leukemia cells. **D.** Correlation between inhibition of p-eIF4E and inhibition of cell proliferation by HHT (*R*^2^ = 0.87). The inhibitory rates of p-eIF4E by HHT at various concentrations were calculated through determining the ratios of the gray area of Western blotting bands between control and HHT-treated groups. **E.** Correlation between inhibition of p-eIF4E and inhibition of cell proliferation by analogs of HHT (*R*^2^ = 0.98). Leukemia cells were treated with HHT at indicated concentrations for indicated times and then collected for analyses of p-eIF4E, t-eIF4E, p-Mnk-1, or Mnk-1 by Western blotting and cell viability by MTT. GAPDH was used as loading control.

To determine whether HHT specifically affected p-eIF4E in AML cells, we examined its effects on p-eIF4E and t-eIF4E, respectively, using Western blotting. Treatment of THP-1 leukemia (AML-M5) cells with HHT for 24h led to a dose-dependent inhibition of p-eIF4E (Fig. 1A). In contrast, t-eIF4E level was not affected under these conditions (Fig. 1A). Similar results were observed in primary AML cells (Supplementary Fig. S1C–S1E). It turned out that the effects of HHT on p-eIF4E level were time dependent (Fig. 1B). To investigate whether HHT had off-target effects that could inhibit other signaling molecules, we tested its ability to inhibit MNK1, a direct upstream kinase that phosphorylates eIF4E (19) and Mcl-1, a downstream oncoprotein of p-eIF4E. At concentrations that significantly inhibited phosphorylation of eIF4E, HHT had no obvious effect on the phosphorylation of MNK1 (Fig. 1C), whereas Mcl-1 level exhibited a parallel decrease with p-eIF4E (Fig. 1A), indicating that the observed decrease in p-eIF4E levels is not a consequence of nonspecific toxicity. These results strongly suggest that HHT specifically reduces p-eIF4E and its downstream target Mcl-1.

To assess whether HHT-mediated reduction of p-eIF4E was associated with its anti-leukemia activity, THP-1 cells were treated with HHT at indicated concentrations for 48 h and then collected for analyses of cell viability with MTT and p-eIF4E levels with Western blotting. We observed that HHT-mediated anti-leukemia activity was positively correlated with p-eIF4E inhibition (*R*^2^ = 0.87) (Fig. 1D). To further confirm these observations, we next selected six HHT analogs with different IC_50_ for inhibition of cell proliferation and then determined their anti-proliferative activity and inhibitory effect on p-eIF4E of AML cells, respectively (Supplementary Table S1). There is also a significant correlation (*R*^2^ = 0.98) between IC50 values of the analogs for leukemia cell proliferation and inhibition of p-eIF4E (Fig. 1E). These results strongly suggest that HHT might be a specific p-eIF4E antagonist that inhibits leukemia cell growth.

### HHT binds to and decreases nuclear p-eIF4E in leukemia cells

**Figure 2:**
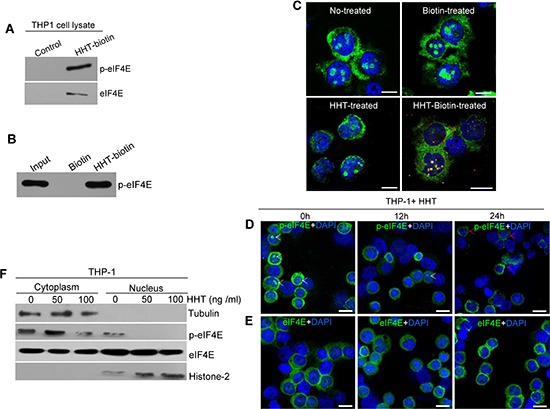
HHT binds to and decreases nuclear p-eIF4E in leukemia cells **A.** HHT interacts with p-eIF4E and t-eIF4E in THP-1 cell lysates. Cell lysates were incubated with HHT-biotin or biotin only (Control), and then co-precipitated with streptavidin agarose resin. p-eIF4E and t-eIF4E in co-precipitated complexes were detected by Western blotting with antibodies against t-eIF4E or p-eIF4E. **B.** Pull-down of purified p-eIF4E protein with HHT-biotin. Purified p-eIF4E were incubated with HHT-biotin followed by precipitation with streptavidin agarose resin. Precipitated complexes were separated by SDS-PAGE, transferred to nitrocellulose membranes, and probed with antibody against p-eIF4E. **C.** Confocal images of HHT colocalization with nuclear p-eIF4E in THP-1 cells. THP-1 cells were treated with HHT-biotin or HHT or biotin for 12 h before analysis by immunofluorescence staining. Red, HHT-biotin; green, p-eIF4E; blue: cell nuclei. The scale bars were 10 μM. **D.** and **E.** HHT reduced the levels of p-eIF4E in the nucleus but did not affect total eIF4E in THP-1 cells. THP-1 cells were treated with HHT (100 ng/ml) for 24 h before analysis of p-eIF4E and t-eIF4E levels by immunofluorescence staining. Green, p-eIF4E or t-eIF4E; blue, cell nuclei. The scale bars were 20 μM. **F.** Western blot analyses of p-eIF4E and t-eIF4E levels in THP-1 cells treated with HHT at the indicated concentrations for 72 h. Tubulin and histone were used as cytoplasm and nucleus protein loading controls, respectively.

To confirm above observations, we next used biotin-labeled HHT (HHT-biotin) to capture p-eIF4E protein in leukemia cells. Whole cell lysates of THP-1 cells were incubated with HHT-biotin and then precipitated by streptavidin resin. After extensive washing, the precipitated complexes were used for analyses of p-eIF4E and t-eIF4E. As shown in Fig. 2A, both p-eIF4E and t-eIF4E were detected by Western blotting. Consistent with this, p-eIF4E protein band (arrow indicated) was revealed with SDS-PAGE and coomassie blue staining assay (Fig. S2), and confirmed by Western blotting (Fig. 2A) and mass spectroscopic analysis. To examine whether purified p-eIF4E protein interacted with HHT, we carried out pull-down assay using p-eIF4E and found that HHT-biotin bound to p-eIF4E (Fig. 2B). These results indicate that HHT can directly bind to p-eIF4E protein.

To reveal where HHT bound to p-eIF4E in leukemia cells, THP-1 cells were treated with HHT-biotin or biotin or HHT for 12 h and then collected to analyze the co-localization of HHT-biotin using immunofluorescence staining with p-eIF4E antibody. We observed that HHT-biotin was present throughout the cytoplasm and nuclei but predominantly co-localized with nuclear p-eIF4E (Fig. 2C). To verify this observation, THP-1 cells were treated with HHT (100 ng/ml) for 12 h or 24 h, after which the distributions of p-eIF4E and t-eIF4E were evaluated by immunofluorescence staining. P-eIF4E levels in the nucleus were greatly reduced after 12 h of incubation with HHT and disappeared after 24 h (Fig. 2D), but t-eIF4E was not obviously affected (Fig. 2E). Interestingly, we observed that HHT promoted the aggregation of nuclear p-eIF4E in the perinuclear space and cytoplasm, concurrent with the disappearance of nuclear p-eIF4E (Fig. 2D, red arrows). Western blot analysis also showed that HHT treatment caused a marked decrease in nuclear p-eIF4E level in THP-1 (Fig. 2F). These results indicate that HHT primarily targets p-eIF4E, especially nuclear p-eIF4E, in leukemia cells.

### The phosphorylated Ser209 residue of p-eIF4E is critical for HHT binding

To reveal the potential binding sites of p-eIF4E for HHT, HHT molecule was initially docked to eIF4E protein (PDB code 1wkw) by implementing our in-house developed All-Around Docking (AAD) methodology (25). AAD allows a small molecule to search the whole surface of the target protein for the binding site that has the lowest docking score by utilizing Schördinger Glide (26) as docking software. Then three types of molecular dynamics (MD) simulations were performed by using NAMD softeware (27) based on the initial binding complex of eIF4E/HHT: wild-type (eIF4E), phorsphorylated S209 (p-eIF4E) and S209-deletion eIF4E-Δ209S, respectively. Each MD simulation runs 25ns with a 1fs time step, 15 layers of water molecules and 0.5M NaCl added to periodic water box, Particle Mesh Ewald method to treat long-range charge interaction, and a 12Å cutoff for van der Waals interactions.

**Figure 3:**
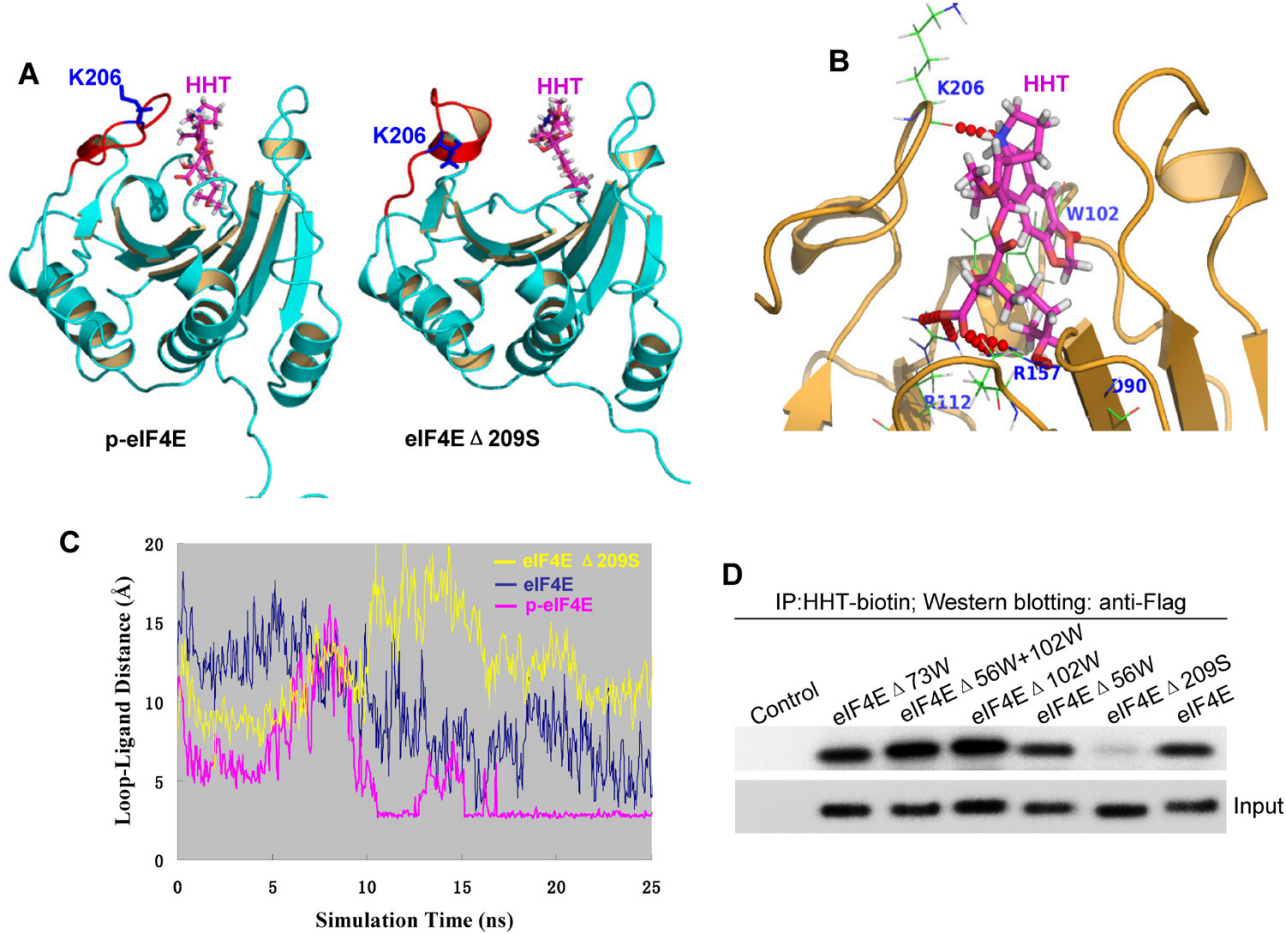
The phosphorylated Ser209 residue of p-eIF4E is critical for HHT binding **A.** Binding mode of HHT in complex with p-eIF4E (left panel) and eIF4EΔ209S (right panel) from 25ns snapshot of MDS. **B.** A hydrogen bond between K206 backbone atom O and HHT molecule was formed. **C.** Minimum distance comparisons of loop region of 201–212a.a. to HHT molecule during 25ns molecular dynamics simulations. Three types of simulations were carried out: wild-type eIF4E/HHT complex simulation is depicted as blue color, phosphorylated S209 simulation is shown as pink color, and eIF4EΔ209S is as yellow color. **D.** Effects of eIF4E mutations on binding of eIF4E to HHT. HEK 293 cells were transfected with indicated FLAG-eIF4E deletion mutants and incubated with biotin-HHT (100 ng/ml) for 12 h. Cell lysates were subjected to streptavidin agarose affinity assay, followed by western blot analysis with anti-FLAG or anti-eIF4E antibodies.

We found that the flexible loop ranging from 201a.a to 212a.a. (Fig. 3A, red) was the key region of eIF4E for the binding of HHT. The snapshots of 25 ns simulations for phorsphorylated S209 (p-eIF4E) and S209-deletion (eIF4E-Δ209S) were shown in Figure 3A. We observed that the loop region in p-eIF4E (Fig. 3A, left) shifted toward HHT molecule to gain stronger protein-ligand interaction. A hydrogen bond between K206 backbone atom O and HHT molecule was formed (Fig. 3B). On the other hand, the loop region in eIF4E-Δ209S (Fig. 3A, right) lost its flexibility due to the formation of a local helix. Thus its binding to HHT molecule would be much weaker. This could be further explained by checking the minimum distance of the loop region to HHT molecule as shown in Fig. 3C. During p-eIF4E simulation, this loop region was flexible and eventually formed a close and stable interaction with HHT molecule with minimum distance of about 2.8Å after 17 ns simulation, as shown in pink color in Figure 3C. However, for eIF4E-Δ209S simulation as displayed in yellow color, the loop region was always more than 8Å away from HHT molecule. For eIF4E (blue-color), no stable interaction was observed as well. The simulation results suggest that HHT should have much stronger binding affinity to p-eIF4E protein than to eIF4E-Δ209S protein.

To identify any eIF4E amino acid that was bound by HHT, we generated a series of eIF4E mutants, including W56, W73, W102 and Ser209 deletion mutants, and then determined the ability of HHT to bind to these mutants in HEK293 cells. W73 is related to its ubiquitination/degradation, W56 and W102 are involved in its cap binding activity, and S209 is the phosphorylation site. Mutation of Ser209 abolished the binding of eIF4E to HHT, which is consistent with above computer modeling results, whereas mutation of other amino acids, including eIF4EΔ56W, eIF4EΔ73W and eIF4EΔ102W, did not affect the binding of eIF4E to HHT (Fig. 3D). These results show that the phospho Ser209 residue of p-eIF4E is critical for HHT binding and binding affinity of HHT to p-eIF4E is higher than to eIF4E.

### HHT promotes proteasome-dependent degradation of p-eIF4E via enhancing its SUMOylation

**Figure 4:**
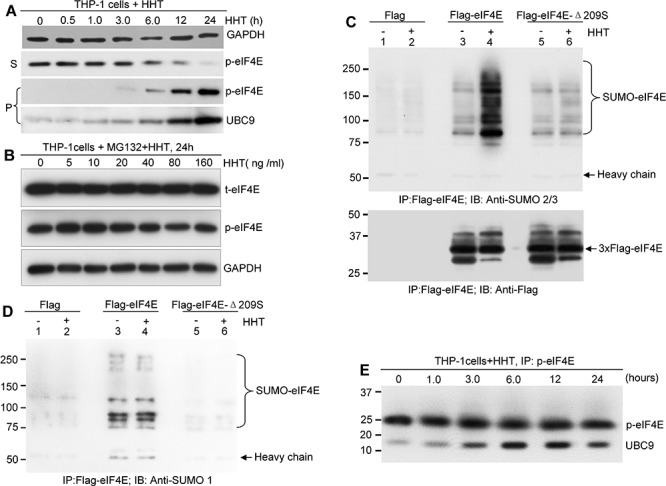
HHT promotes proteasome-dependent degradation of p-eIF4E via enhancing its SUMOylation **A.** Western blot analysis of p-eIF4E in the supernatant (S) and pellet (P) fractions of lysates of THP-1 cells treated with 100 ng/ml HHT for the indicated time points. GAPDH was used as loading control. **B.** Western blot analysis of p-eIF4E and total-eIF4E (t-eIF4E) in whole cell lysates from THP-1 cells treated with the indicated concentrations of HHT and MG132 (2 μg/ml) for 24 h. **C.** and **D.** HEK 293 cells transfected with FLAG-eIF4E or FLAG-eIF4E-209 plasmids were treated with or without HHT for 12 h. After harvesting, cells were lysed, followed by immunoprecipitation with anti-FLAG M2 beads and subsequent western blot analysis with either anti-SUMO2/3 (C) or anti-SUMO1 (D). **E.** Time course analysis of HHT-induced UBC9 conjugation to p-eIF4E in THP-1 cells. THP-1 cells were treated with HHT (100 ng/ml) for the indicated time points.

It has been shown that eIF4E is a SUMO substrate (28). To determine whether HHT-mediated decrease of p-eIF4E is involved in SUMOylation modification, we evaluated the effects of HHT on both p-eIF4E and the small ubiquitin-like protein modifier (SUMO)-conjugating enzyme UBC9 in leukemia cells. In as little as 3 h of exposure, HHT induced a shift of p-eIF4E from the supernatant (S) of cell lysates to the detergent-insoluble pellet (P). This shift was associated with a concomitant increase level of UBC9 (Fig. 4A). After 24 h of HHT exposure, most p-eIF4E was present in the detergent-insoluble pellet (Fig. 4A), suggesting that HHT increased the levels of denatured p-eIF4E in leukemia cells. These results are consistent with our observations that HHT induced p-eIF4E aggregation in the perinuclear space and cytoplasm (Fig. 2D, red arrows). Furthermore, we found that the proteasome inhibitor MG132 strongly blocked the HHT-induced decrease in p-eIF4E levels in leukemia cells (Fig. 4B), suggesting that HHT treatment triggers the proteasome-dependent degradation of p-eIF4E protein.

We next investigated whether HHT denatured the p-eIF4E by increasing its SUMOylation. HEK 293 cells were transfected with plasmids expressing FLAG-tagged eIF4E or FLAG-tagged eIF4E-Δ209S, and then treated with or without HHT for 12 h. Cell lysates were immunoprecipitated with anti-FLAG M2 beads and subsequently probed with anti-SUMO1, anti-SUMO2/3, or anti-Flag antibodies during western blot analysis. We detected multiple bands larger than the 25 kDa band of wild-type eIF4E (Fig. 4C, lane 4) but did not detect the eIF4E-Δ209S mutant (Fig. 4C, lane 6) in HEK293 cells with anti-SUMO2/3 antibody after HHT treatment. This result could not be repeated with the anti-SUMO1 antibody (Fig. 4D). Moreover, treatment with HHT (100 ng/ml) increased the amount of SUMOylated p-eIF4E oligomerization (Fig. 4C, lane 4). These results indicate that HHT treatment enhances SUMO2/3-mediated SUMOylation of p-eIF4E but not t-eIF4E. In addition, phosphorylation of Ser209 is required for HHT-mediated SUMOylation of p-eIF4E (Fig. 4C, lane 6). To further confirm these observations, we performed a time course analysis of HHT-induced UBC9 conjugation to p-eIF4E in leukemia cells. HHT increased the binding of UBC9 to p-eIF4E in a time-dependent manner (Fig. 4E). These results indicate that HHT induces SUMO2/3-mediated SUMOylation of p-eIF4E protein by enhancing the binding affinity of UBC9 for p-eIF4E, which in turn might lead to proteasome-dependent degradation of p-eIF4E. Therefore, we propose a working model for HHT-induced degradation of p-eIF4E: HHT binds to the loop of 201–212aa of p-eIF4E and causes conformational changes, which facilitate oligomerization and SUMOylation of p-eIF4E and ultimately lead to its proteasome-dependent degradation.

### HHT eradicates human AML-M5 expressing high level of p-eIF4E in orthotopic mouse model

**Figure 5:**
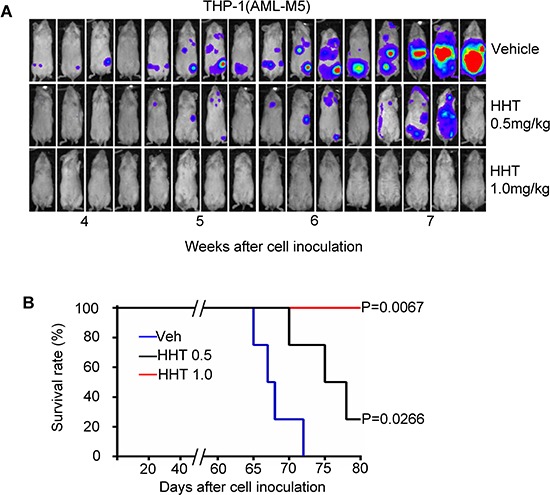
HHT eradicates human AML-M5 expressing high level of p-eIF4E in orthotopic mouse model **A.** Xenogen images of NSG mice injected with 100,000 luciferase-labeled THP-1 cells and then treated with HHT (0.5 or 1 mg/kg body weight) or vehicle control by intraperitoneal injection for 14 consecutive days. **B.** Kaplan-Meier survival analysis of mice treated with HHT. Blue, vehicle control (Veh); black, HHT 0.5 mg/kg body weight; red, HHT 1.0 mg/kg body weight.

To gain *in vivo* evidence that HHT can eradicate AML cells expressing high level of p-eIF4E, we established an aggressive subtype of AML-M5 orthotopic mouse model. To do this, we engrafted NSG mice with THP-1 cells, which express high levels of p-eIF4E. Leukemic mice were intraperitoneally injected with HHT (0.5 or 1.0 mg/kg body weight) daily for 14 consecutive days. Consistent with the *in vitro* results, treatment with HHT greatly decreased the leukemia burden (Fig. 5A) and increased survival time (Fig. 5B) in a dose-dependent manner. Unexpectedly, treatment with the higher dose of HHT (1.0 mg/kg body weight) completely eradicated AML-M5 *in vivo* and all the mice were disease-free survival on day 80 (Fig. 5). These results suggest that targeting p-eIF4E by HHT has potential to eradicate AML expressing high level of p-eIF4E.

## DISCUSSION

In this study, we demonstrate that it is possible to directly target p-eIF4E, an unrealized attractive target for anti-tumor intervention, using small molecules. The observation that HHT, a widely marked anti-cancer agent (28–30), specifically antagonizes p-eIF4E function and inhibits growth of AML cells expressing high levels of p-eIF4E by targeting the phosphorylated serine 209 residue of p-eIF4E, but not for eIF4E, for SUMO2/3-mediated degradation is somewhat unexpected and of particular interest.

A striking finding of this study is that HHT might be the first-in-class p-eIF4E-targeted drug. Computer modeling results show that HHT molecule could form a close and stable interaction with p-eIF4E with minimum distance of about 2.8Å but not with t-eIF4E, which is further confirmed by eIF4E mutation analysis. These results indicate that the binding affinity of HHT to p-eIF4E is higher than that to eIF4E. We also found that HHT preferentially targets the loop of 201–212aa of p-eIF4E but not of t-eIF4E, which in turn induces oligomerization, SUMO2/3-mediated SUMOylation and subsequent proteasome-dependent degradation of p-eIF4E proteins. Although precise mechanism by which HHT selectively induces degradation of p-eIF4E is unclear, the close and stable interaction between HHT and p-eIF4E might alter the conformation of p-eIF4E and increase the binding affinity of UBC9 for p-eIF4E, which in turn lead to enhanced SUMOylation of p-eIF4E. Importantly, compound structure-activity relationship (SAR) analysis results showed that HHT-mediated anti-leukemia activity is positively correlated with p-eIF4E levels. These findings are of great significance because t-eIF4E activity is required for proliferation of both tumor and normal cells (7–9), whereas p-eIF4E is not essential for normal cell proliferation and survival, but is specifically required for cancer cells (10, 13, 14). Notably, we observed that HHT-mediated degradation of p-eIF4E greatly decreases its target molecule Mcl-1, a critical regulator that promotes the survival of AML stem cell (31), implying that targeting p-eIF4E by HHT might be an ideal therapeutic approach to eliminate leukemia stem/progenitor cells of AML. However, considering the frequent adverse effects of HHT on the hematological system, gastrointestinal tract and other organs (29, 30, 33), and because HHT also inhibits other signaling molecules such as p-210Bcr-Abl (24), c-myc (22), Mcl-1 (23), we can not rule out the possibility that HHT may have additional targets that are involved in both of anti-tumor activity and cytotoxicity.

As expected, we found that HHT potently inhibited the growth of AML cells expressing high levels of p-eIF4E *in vitro* and *in vivo*. Unlike ribavirin, a broad-spectrum antiviral drug that blocks eIF4E activity through a physical mimic of the m7G cap, exhibited mild anti-tumor activity (16, 32), treatment with HHT as a single agent efficiently inhibited the growth of THP-1 leukemia cells expressing high levels of p-eIF4E, an aggressive M5 subtype of acute myeloid leukemia (AML-M5), in NSG mice and greatly increased the survival time of leukemia mice. Surprisingly, HHT with the higher dose (1.0 mg/kg body weight) completely eradicated AML-M5 *in vivo* and all the mice obtained long-term disease-free survival.

It is tempting to speculate that this unique inhibition effect of HHT on p-eIF4E may be further exploited for the development of new anticancer modalities. The pharmacological properties of this natural compound can be optimized and improved by analyzing the structure-function relationships governing inhibition of p-eIF4E and target selectivity. In addition, these pharmacological probes of p-eIF4E can provide important new insights into the function of p-eIF4E and its mechanism by which p-eIF4E specifically regulates the growth of cancer cells.

## MATERIALS AND METHODS

### Detection of binding of HHT to p-eIF4E and eIF4E

Lysis buffer: 50 mM Tris-HCl, pH6.8, containing 50 mM Tris/150 mM NaCl/1 mM sodium vanadate/1 mM sodium fluoride/1 mM PMSF/1.0% NP-40, plus protease inhibitors and phosphatase inhibitors. THP-1 cells (1 × 10^8^) were lysed with lysis buffer (2 ml) for 15 min on ice. Cell lysate supernatants were collected by centrifugation (14,000 × g, 15 min, 4°C) and split in two aliquots (1 ml/aliquot). One aliquot was precipitated with 50 μg of HHT-biotin while the other aliquot was precipitated with biotin (negative control) overnight at 4°C. The structure of HHT-biotin and its preparation procedure were presented in Fig. S3. After incubation with 100 μl of Streptavidin agarose resin for 1 h at 4°C with continuous inversion, the precipitated complexes were pelleted. After washed five times with lysis buffer, the precipitated complexes were incubated at 95°C for 10 min and then loaded and separated on a 10% SDS-PAGE for analyses of p-eIF4E and t-eIF4E by Western blotting, and protein profile staining with coomassie brilliant blue staining Kit (Invitrogen, Grand Island, NY). eIF4E identification was achieved by mass spectroscopic analysis.

### p-eIF4E pull down assay

Purified p-eIF4E protein (1.5μg) was incubated with HHT-biotin (100 μg) for 4h at 4°C. After incubation with Streptavidin agarose (100μl) (Thermo, Waltham, MA) resin for 0.5 h at 4°C with continuous inversion, the complexes were pelleted and washed five times with lysis buffer. The complexes were incubated (95°C, 10 min) and separated on a 10% SDS-PAGE, and detected by Western blot analysis for p-eIF4E.

### Western blot analysis

Cells were washed twice with 1 × phosphate-buffered saline (PBS, pH 7.2), and total cellular protein was extracted with RIPA buffer. Protein samples were subjected to SDS-PAGE (12% polyacrylamide gels), and then transferred to nitrocellulose membranes (Whatman, Pittsburgh, PA), blocked with 5% nonfat milk in TBS-Tween 20 (TBS-T) and incubated with primary antibodies overnight at 4°C. After three washes with TBS-T, membranes were probed with a horseradish peroxidase–conjugated secondary antibody (Cell Signaling, Beverly, MA) for 1 h at room temperature, and signals were detected by chemiluminescence (Super Signal West Pico; Thermo, Waltham, MA). The antibodies used for western blot analysis were as follows: eIF4E, p-eIF4E, Mcl-1, GAPDH, MNK-1, p-MNK-1, β-actin, histone-2, UBC9, SUMO-1, SUMO-2/3, GST, and Flag antibodies. All antibodies except p-eIF4E antibody (Abcam, Cambridge, MA) were purchased from Cell Signaling Technology (Beverly, MA).

### Immunofluorescence assay

Cells were fixed with freshly prepared 4% paraformaldehyde in PBS for 30 min at room temperature on slides. Cells were then permeabilized with 0.2% Triton X-100 in PBS (pH 7.2) for 4 min and blocked with PBS containing 5% BSA for 30 min at room temperature. Staining of cells with FITC mouse anti-eIF4E antibody (BD Biosciences, 1:100 dilution) was performed overnight at 4°C in PBS containing 1% BSA. For Phospho-eIF4E staining, cells were incubated with rabbit polyclonal anti-phospho-eIF4E antibody (Abcam, Cambridge, MA) overnight at 4°C, and then with a Dylight 488 goat anti-rabbit polyclonal antibody (Abcam, Cambridge, MA) for 1 h at room temperature. After three washes with PBS (pH 7.2), the slides were mounted in Vectashield with DAPI (4′,6′-diamidino-2-phenylindole; Vector Laboratories, Burlingame, CA) and sealed. Fluorescence was observed with a Zeiss Confocal Laser Scanning Microscope.

### Co-localization of HHT and p-eIF4E

THP-1 cells were incubated with HHT-biotin or biotin or HHT for 12 h. Cells were then fixed and permeabilized as described above. Slides were washed twice with 1 × PBS, and incubated with rabbit polyclonal anti-phospho-eIF4E antibody (Abcam, Cambridge, MA) overnight at 4°C. After three washes with 1 × PBS, a Dylight 488 goat anti-rabbit polyclonal antibody (Abcam, Cambridge, MA) was added as the secondary antibody. After two washes with 1 × PBS, slides were incubated with 4μg/ml rhodamine-conjugated streptavidin in PBS (pH 7.2) for 2 h in a humid environment at room temperature. After three washes with PBS (pH 7.2), slides were mounted in Vectashield with DAPI (4′,6′-diamidino-2-phenylindole; Vector Laboratories, Burlingame, CA) and sealed. Fluorescence was observed with a Zeiss Confocal Laser Scanning Microscope.

### Streptavidin agarose affinity assay

EIF4E cDNA was cloned into pIRESneo-3XFLAG vector, with a FLAG tag at its N-terminus. Using this FLAG-eIF4E plasmid, different mutation plasmids were constructed using the QuikChange^®^ II XL Site-Directed Mutagenesis Kit (Agilent, Santa Clara, CA): FLAG-eIF4E-Δ56 (W56 deletion), FLAG-eIF4E-Δ73 (W73 deletion), FLAG-eIF4E-Δ102 (W102 deletion), FLAG-eIF4E-Δ56/102 (W56 and W102 deletion), and FLAG-eIF4E-Δ209 (S209 deletion). HEK 293T cells transfected with FLAG-eIF4E, FLAG-eIF4E-Δ73, FLAG-eIF4E-Δ209, FLAG-eIF4E-Δ102, FLAG-eIF4E-Δ56/102, FLAG-eIF4E-Δ56 or empty vector were treated with 100 ng/ml HHT-biotin for 12 h, and then lysed in RIPA buffer. Cell lysates were incubated with streptavidin agarose (Thermo, Waltham, MA) overnight at 4°C. After three washes with washing buffer (Thermo, Waltham, MA), streptavidin agarose beads were resuspended in 2 × SDS-PAGE loading buffer. The eIF4E input was detected with anti-FLAG antibody (Sigma, St. Louis, MO).

### AML cell line and culture

Human AML-M5 cell line THP-1 was used in this study. Cells were cultured in RPMI-1640 supplemented with 10% fetal calf serum at 37°C in a 95% air, 5% CO2 humidified incubator.

### Human primary leukemia cell samples

Human primary leukemia cell samples were obtained from leukemia patients with their informed consent in accordance with the Declaration of Helsinki. All experiments were approved by the ethics committee of Second Affiliated Hospital, School of Medicine, Zhejiang University.

### Orthotopic model for AML and Xenogen imaging

All animal procedures were approved by the Institution's Ethics Committee. To establish orthotopic model, human AML THP-1 cells were stably transduced with a lentiviral firefly luciferase. Cells (1 × 10^5^) were injected through the tail vein into NSG (NOD/SCID/IL2Rγ−/−) mice. Three days after injection, HHT (0.5 or 1 mg/kg body weight) or vehicle control was intraperitoneally injected for 14 consecutive days. Bioimaging of mice was performed at different time points using an *in vivo* IVIS 100 bioluminescence/optical imaging system (Xenogen, Alameda, CA). Briefly, mice were intraperitoneally injected with D-Luciferin (2.5 mg per mouse) (Promega, Madison, WI) dissolved in PBS 15 min before measuring the luminescence signal. General anaesthesia was induced with 5% isoflurane and continued during the procedure with 2% isoflurane introduced through a nose cone.

### Immunoprecipitation assay

Immunoprecipitation by anti-FLAG M2 beads was done using the FLAG immunoprecipitation Kit (Sigma, St. Louis, MO). After treatment, cell protein was extracted with IP lysis buffer (Thermo, Waltham, MA). Cell lysate was then incubated with pre-washed anti-FLAG M2 beads (50 μl) overnight at 4°C with gentle rotation. Immunoprecipitated complexes were collected by centrifugation (7,000 × g, 1 min, 4°C) and washed three times with 1 ml washing buffer by resuspension and centrifugation (7,000 × g, 1 min, 4°C). The immunoprecipitate was detected by Western blot analysis.

### Statistical analysis

All statistical analyses were performed with Prism GraphPad. Results are expressed as means ± SEM. Differences were evaluated by t test analysis of variance and P values less than 0.05 were considered statistically significant.

## SUPPLEMENTARY FIGURES AND TABLE


