# Supplementary material for: Small-molecule induction of phospho-eIF4E sumoylation and degradation via targeting its phosphorylated serine 209 residue
Source: Oncotarget. 2015 Apr 10;6(17):15111–21. doi: 10.18632/oncotarget.3615 (PMC4558139; doi:10.18632/oncotarget.3615)
Supplement: Supplementary file 1 [file oncotarget-06-15111-s001.pdf]

SUPPLEMENTARY FIGURES AND TABLE

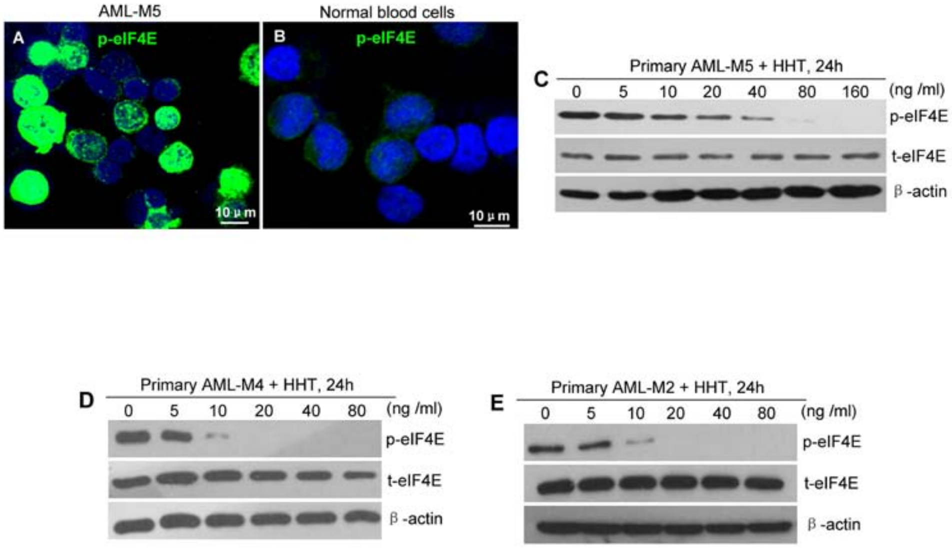

Supplementary Figure S1: p-eIF4E is highly expressed in primary leukemia cells of AMLpatients and reduced by HHT. (A–B) Images of immunofluorescence staining of p-eIF4E from primary AML-M5 cells **A.** and normal blood cells **B.** Green: p-eIF4E. Blue: cell nucleus visualized by DAPI. **(C–E)** HHT selectively reduced p-eIF4E in a dose-dependent manner but did not affect t-eIF4E of AML primary leukemia cells. Primary leukemia cells were treated with HHT at indicated concentrations for 24 h and then collected for analyses of p-eIF4E and t-eIF4E. β-actin was used as loading control.

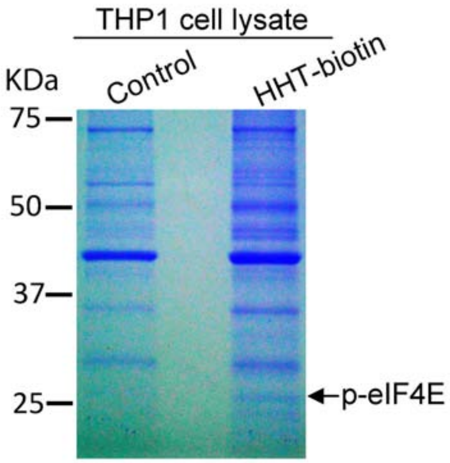

Supplementary Figure S2: Identification of p-eIF4E protein that interacts with HHT. Cell lysates from THP1 cells were incubated with HHT-biotin or biotin followed by co-precipitated with streptavidin agarose resin. Co-precipitated complexes were separated by SDS-polyacrylamine gel electrophoresis (SDS-PAGE) and coomassie brilliant blue staining. The identification of eIF4E was achieved by mass spectroscopic analysis.

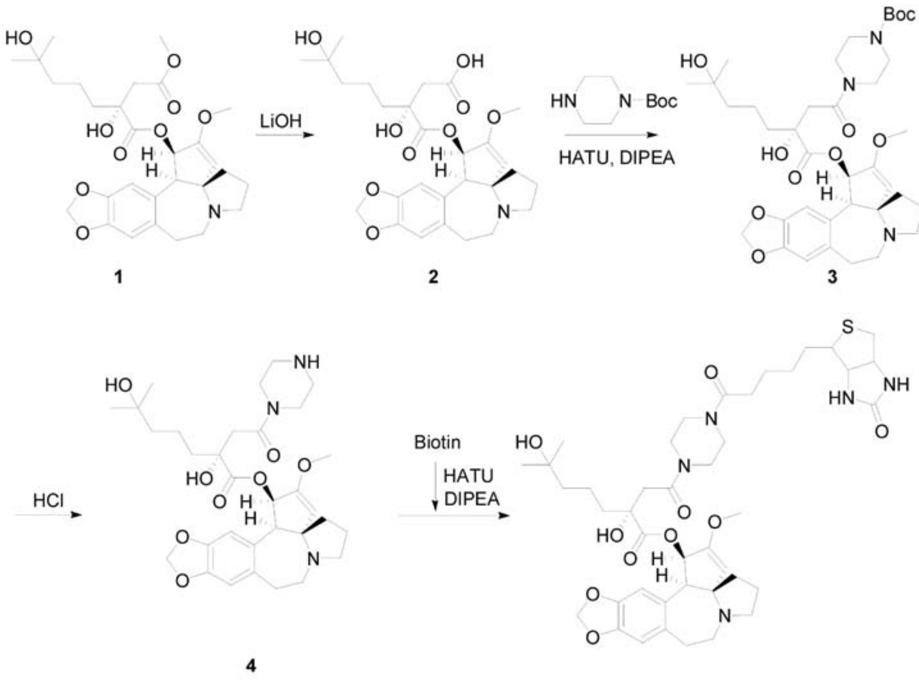

Supplementary Figure S3: The structure of HHT-biotin and its preparation procedure.

Supplementary Table S1. Bioactivities of HHT and its analogs

Entry	Structure	IC50 (ng/ml)	
		p-eIF4E level	THP-1 proliferation
HHT<br>$C_{29}H_{39}NO_9$<br>MW:545.62	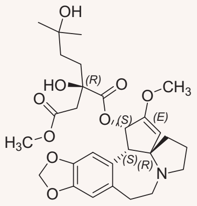	64.54	9.72
HT<br>$C_{28}H_{37}NO_9$<br>MW:545.62	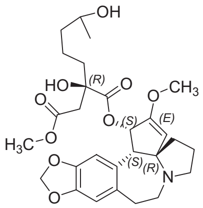	113.36	24.06
H0722<br>$C_{39}H_{45}N_3O_{11}S_2$<br>MW:795.9	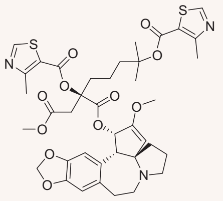	306.55	460.8
H021<br>$C_{33}H_{46}N_2O_9$<br>MW:614.7	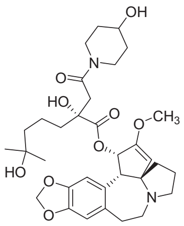	1280.93	7466.74
H0732<br>$C_{43}H_{47}NO_{11}$<br>MW:753.8	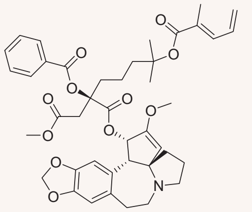	804.72	3284.59
H025<br>$C_{33}H_{46}N_2O_9$<br>MW:614.7	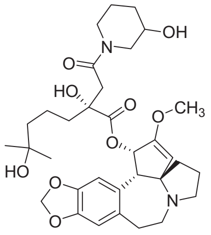	676.43	5434.63
